# Associations between screen viewing at 2 and 3.5 years and drawing ability at 3.5 years among children from the French nationwide Elfe birth cohort

**DOI:** 10.1038/s41598-023-50767-0

**Published:** 2024-01-03

**Authors:** Lorraine Poncet, Mélèa Saïd, Shuai Yang, Falk Müller-Riemenschneider, Claire Berticat, Michel Raymond, Mélissa Barkat-Defradas, Marie-Aline Charles, Jonathan Y. Bernard

**Affiliations:** 1 Centre for Research in Epidemiology and Statistics (CRESS), Université Paris Cité, Université Sorbonne Paris Nord, Inserm, INRAE, 75004 Paris, France; 2 Saw Swee Hock School of Public Health, National University of Singapore, Singapore, Singapore; 3 Berlin Institute of Health, Charite University Medical Centre, Berlin, Germany; 4 ISEM, CNRS, EPHE, IRD, University of Montpellier, 34095 Montpellier, France; 5 Unité mixte Elfe, Ined, Inserm, EFS, 93322 Aubervilliers, France; 6 Singapore Institute for Clinical Sciences (SICS), Agency for Science, Technology and Research (A*STAR), Singapore, Singapore

**Keywords:** Epidemiology, Paediatric research

## Abstract

The effect of screen viewing on children’s cognitive development has been of concern among parents and researchers. This study investigated the association between children screen time, as reported by parents, and drawing ability, and the confounding effects of socioeconomic characteristics (such as parental education, household income, migration status) and children’s competing activities (such as drawing practice, extracurricular activity, outdoor time, sleep time, time playing with parents). Participants included 7577 children aged 3.5 years (50% girls) who underwent the Draw-a-person test (McCarthy score [range = 0–12 points]) in the French nationwide Elfe birth cohort, initiated in 2011. Sex-stratified zero-inflated Poisson regression models were used. Increased screen time was associated with a higher likelihood to obtain a null score in boys (OR 1.15, 95% CI 1.07–1.23) and girls (1.13 [1.03–1.24]) and a lower score in girls only (β =  − 0.02, 95% CI − 0.04; − 0.01). After adjusting for SES, associations were no longer observed, indicating that the association between screen time and drawing abilities was confounded by socioeconomic characteristics.

## Introduction

Screen viewing has dramatically evolved in the last decade, with content, uses and devices multiplying rapidly. As media exposure has increased, screen viewing in young children has raised concern, with the fear that screen viewing might impede their cognitive development. Studies have displayed mixed results on the link between screen viewing and cognitive development in childhood. While excessive time spent viewing TV has been associated with poor attention^1^ and delayed language development^2,3^, it seems that a consistent determinant of cognitive development is the context created by parents around screen viewing^4^. Concerning language development, studies have stressed the importance of co-viewing and discussing content with the child: up to 3 years old, children can learn verbs from videos when accompanied by live social interaction^5^. Discussing content with parents can mitigate the risk of delayed language development associated with screen viewing before school^6^. Media content is also critical: while educational programmes promote language development, age-inappropriate or fast-paced programmes are associated with higher hyperactivity, lower social skills^7^ and poorer executive functioning^8^.

The Draw-a-Person test is a popular tool among clinicians and psychiatrists, where children are asked to draw a person on a blank sheet of paper. The drawing is then scored according to the body parts’ presence and level of detail (depending on the version of the score), with the most elaborate drawings obtaining the highest scores. The test was developed in 1926, originally as a measure of intelligence^9^. However, studies have shown only moderate correlation with other measures of intelligence^10^. At the minimum, the test informs on the child’s fluency with a graphic lexicon of forms or schematic models and a graphic syntax^11,12^. As these elements depend on the child’s cognitive functions^13^, the test can contribute, among other tools, to measure cognitive development. However, other elements also determine the scoring on this test: the child’s fine motor skills^14^, frequency of drawing practice, and level of exposure to culture-specific schematic models^11^.

To our knowledge, only two studies have addressed the relationship between screen viewing and drawing in children using a version of the Draw-a-Person test^15,16^. Winterstein and Jungwirth suggested a strong detrimental effect of screen viewing on the drawing ability of 1859 German children of pre-school age, results that were widely publicised by media outlets. André and Cochetel showed significantly lower drawing skills in 127 French children aged 5–6 years when using screens more than 10 h per week. However, both studies conducted only bivariate analyses, not controlling for the potentially confounding effects of households’ socioeconomic status^12^, calling for more robust analyses of the observed associations.

In this work, we propose two alternative hypotheses concerning the relationship between children’s screen viewing and drawing ability. Our first hypothesis is that the relationship between screen viewing and the Draw-a-Person test score can be explained, partly or fully, by households’ socioeconomic status. Indeed, a negative association between parents’ educational level, income and children’s screen viewing has been widely documented^17–20^. On the other hand, as socioeconomic status is associated with children’s cognitive functioning^21,22^, we expect that children from households with lower socioeconomic status have poorer drawing abilities. Secondly, we hypothesize that children’s screen viewing has a detrimental effect on drawing ability through the displacement of other activities such as drawing or painting practice and other activities and games more beneficial to visual perception and attention, visual-motor coordination, working memory and complex spatial abilities, which can promote drawing skills^13^.

We aimed to assess the associations between screen time and drawing ability, and examine whether these are confounded by household socioeconomic status and children’s activities competing with screen time.

## Methods

### Study sample and participants

We used data from the Elfe birth cohort (Etude Longitudinale Française depuis l’Enfance), a prospective nationally representative birth cohort initiated in 2011 in 349 randomly-selected maternity hospitals. The general objective was to examine the determinants of the child’s development, health and socialization from birth to adulthood. The study design and protocol have been detailed elsewhere^23^. In brief, parents and their new-borns were invited to participate if they met the following inclusion criteria: birth after 33 weeks’ gestation, mothers > 18 years old, not planning to leave France within the next 3 years and with the ability to read and understand French, Arabic, Turkish or English. Participation rate at inclusion was 51% and 18,329 new-borns were included. Baseline characteristics were collected in a face-to-face questionnaire at inclusion, and follow-up surveys were conducted by phone, email or face-to-face interviews depending on the time points. The survey at age 2 years, carried out in 2013, consisted of a phone interview. The survey at age 3.5 years, carried out in 2014–2015, included a phone interview and a home visit.

### Ethics approval and consent to participate

Informed consent was obtained from all participants or their legal guardians. Mothers provided written consent for their own and their child’s participation. Fathers provided written consent for the child’s participation when present at inclusion or were informed about their rights to oppose it. The Elfe study was approved by the Advisory Committee for Treatment of Health Research Information (Comité Consultatif sur le Traitement des Informations pour la Recherche en Santé) under approval numbers 10.623 (10/26/2010) and 13.004 (01/24/2013), the National Data Protection Authority (CNIL) (approval numbers 2011-081, 03/17/2011, and 2013-113, 04/25/2013) and the National Statistics Council. All methods were performed in accordance with the relevant guidelines and regulations.

### Child screen time

We analysed screen time exposure measured at age 2 and 3.5 years. In the phone questionnaires at both time points, parents were asked to report their child’s daily screen time, through questions concerning time spent on different types of screen devices: television, tablet or computer, videogames (on console), smartphone. In the questionnaire at 2 years, parents indicated time spent on each device on a typical weekday and on a typical weekend day: “On a typical weekday/weekend day, how much time in total does your child use the television/tablet or computer/videogames (on console)/smartphone?” For each device, we computed an average daily screen time in hours per day:

$$Average\, daily \,time=(weekday\, time\,\times\, 5+weekend \,time\,\times \,2)/7.$$

In the questionnaire at 3.5 years, parents indicated time spent on each device on a typical weekday, on a typical Saturday and on a typical Sunday. “On a typical weekday/Saturday/Sunday, how much time in total does your child use the television/tablet or computer/videogames (on console)/smartphone?” For each device, we computed an average daily screen time in hours per day:

$$Average\, daily \,time=(weekday\, time\,\times\, 5+Saturday\, time+Sunday \,time)/7.$$

Average daily screen times on each device were added to compute average daily total screen time (hours/day). Screen time variables were used as continuous variables.

Additionally, to consider the potential use of internet, we pooled time spent on device that facilitate access to the internet: tablet, PC and smartphone.

### Draw-a-person test

At age 3.5 years, children underwent the Draw-a-Person test during home visits: they were asked to draw a person, to the best of their ability, taking the time they needed. Drawings were collected and scored using the McCarthy score^24^ adapted by Arden et al. into a 12-point score^25^. The McCarthy Draw-a-person test has been validated in a population of children aged 6 to 8.5 years^26^. Scoring was based on the presence of eleven body parts (head, trunk, arms, hands/fingers, legs, feet, eyes, nose, mouth, ears, hair) and clothing, each of them yielding one point. Scoring was carried out by two independent raters, with an excellent agreement of the two scores (Cohen’s Kappa coefficient: 0.89). As there was no reference rater, the two scores were averaged for improved precision. Children who did not draw anything had missing values for the drawing score. When children drew something else than a person, or when a human figure was not discernible, they were given a score of zero^25^. Surveyors provided indications as to the context of the drawing test: drawing execution and the hand used for drawing.

### Covariates

We used variables informing on household socioeconomic status: maternal and paternal educational level (junior high school, technical high school, high school graduate, 2 years of university, 3–4 years of university, ≥ 5 years of university), household income per consumption unit at 3.5 years in euros (< 1100; 1100–1670; 1670–2700; ≥ 2700), parents living together (yes or no) and household migration status (both parents born abroad, one parent born abroad or both parents born in France).

We used variables informing on child activities likely to compete with screen viewing: extracurricular activities (yes or no), time spent playing outside (in hours per day) and total sleep time (in hours per day, including night and nap time). In France, children generally enter preschool within the year of their third birthday, i.e., between 2.5 and 3.5 years of age. Because drawing is a frequent activity in preschool we hypothesized that children who had been in preschool longer at the time of the home visit would be more skilled at the drawing test. We therefore included time between start of preschool and drawing data collection (in months). We included frequency of drawing or painting practice at 2 years (never; sometimes; often; everyday), as it was not included in the questionnaire at 3.5 years.

In the questionnaire at 3.5 years, parents indicated whether they had engaged in a range of activities with their child in the last month (binary variables: yes/no): paint, draw or colour; tell a story; sing a song or play music; read a book again and let the child find parts of the story; have them count or recite numbers; have them copy letters or words; do a puzzle together). We used principal component analysis to reduce information while accounting for co-dependency between these variables. Parents/child activity components were obtained, and we selected the first component, i.e., parental engagement, explaining 32% of variance, as a covariate in our analyses. This principal component analysis has been described in previous work^27^.

Covariates also included socio-demographic variables associated with child screen viewing: child sex (boy or girl), maternal age at birth (≤ 30 years; 31–40 years; > 40 years), birth rank (first born or later born) and gestational age at birth (in weeks of gestation).

Lastly, we took into account two variables informing on the context of the drawing test, as indicated by surveyors: drawing execution by the child at home visit (right away; surveyor had to insist a little; had to insist a lot) and hand used for drawing (right hand; left hand; both hands).

Directed acyclic graphs were used to help us choose relevant covariates.

### Population selection

**Figure 1:**
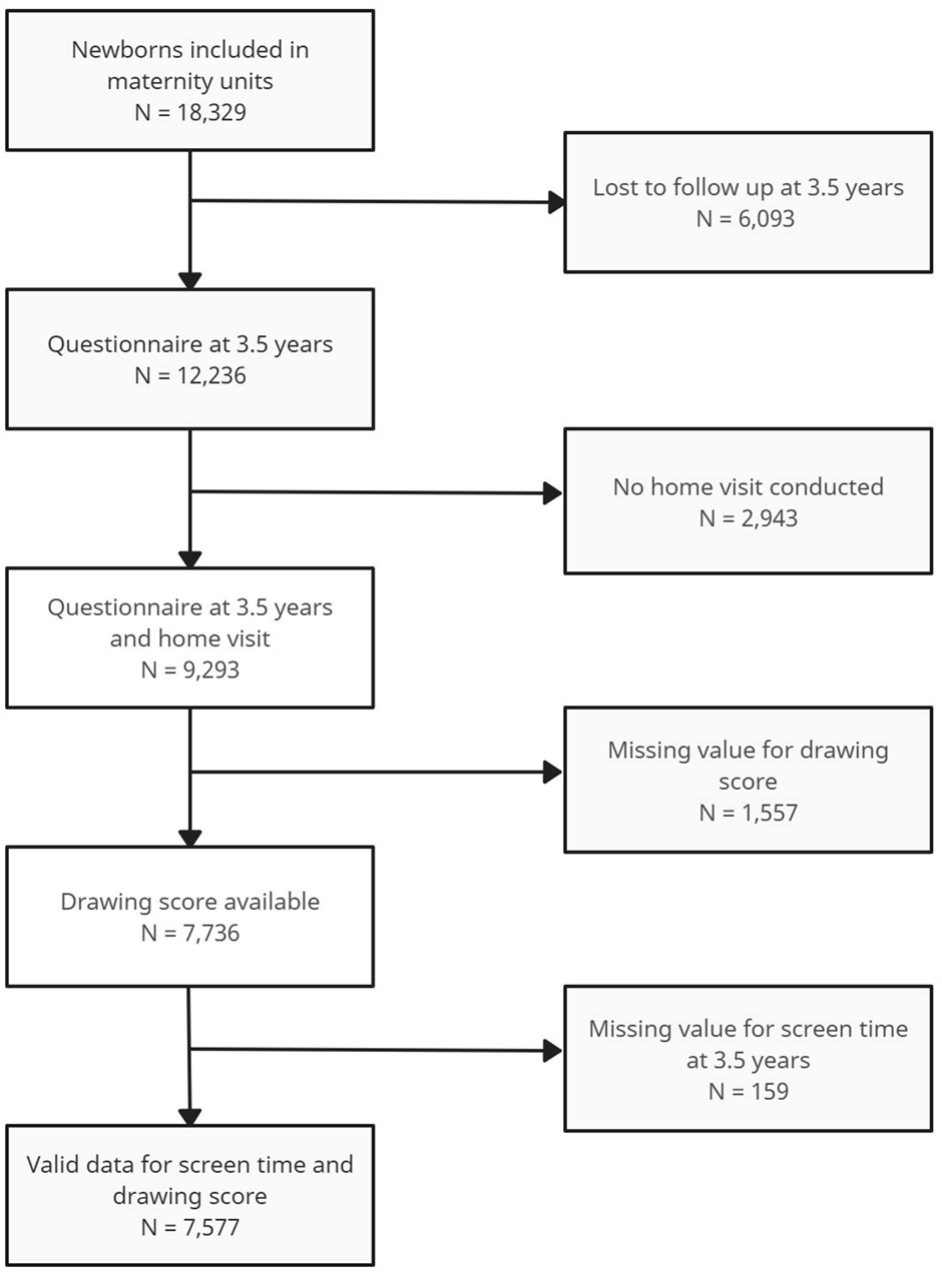
Flowchart of population selection of children with valid data for screen time and McCarthy drawing score at 3.5 years, Elfe birth cohort.

In the survey at age 3.5 years, data were collected on 12,236 children through phone interviews. Among them, 9293 children also agreed to a home visit. Among those visited at home, 7736 children completed the drawing test and had non-missing data for the drawing score. After excluding observations with missing data for screen time (n = 159), our analytical sample included 7577 children with valid data for both screen time and drawing score (Fig. 1).

### Statistical analyses

We presented the distribution of all variables for the analytical sample and the excluded observations. All analyses were stratified according to child sex, as both screen time and McCarthy drawing score differed between boys and girls. Because our outcome variable included a large number of scores equal to zero (26.9% for boys, 13.7% for girls), we used zero-inflated Poisson regression models to examine the associations of total and device-specific screen time variables with the McCarthy drawing score. In brief, this model deals with the inflation of zeros by running in a single step a binary logistic regression explaining the likelihood of obtaining a null score (yes/no), then a linear regression among scores greater than zero. To test our two hypotheses, we constructed two multivariable models: the first model controlled for households’ socioeconomic status, while the second model controlled for the child’s competing activities. Both of these models were also adjusted for socio-demographic variables associated with child screen viewing and on the two variables informing on the context of drawing. A third model adjusted for all variables. To investigate in more detail the role of household socio-economic status on the associations, we stratified analyses on maternal educational level, adjusting for socio-demographic variables and variables informing on the context of the drawing test.

To examine potential collinearity, we tested variance inflation factor in our model; all variance inflation factors were < 1.2, suggesting absence of collinearity.

The analyses were carried out using SAS v9.4 software (SAS Institute Inc, Cary, North Carolina).

## Results

### Population characteristics

The characteristics of the analytical sample and the excluded respondents are shown in Table 1. Among included respondents, two third (62%) of mothers were aged between 31 and 40 years. In both mothers and fathers, 23% had at least 5 years of university. Only 4% of households had two immigrant parents. The mean (± SD) McCarthy score was 4.8 (3.0) points. Child mean total screen time at 2 years was 0.7 (0.9) hours per day, largely consisting of TV time (mean = 0.6, SD = 0.8). Child mean total screen time at 3.5 years was 1.1 (0.97) hours per day, with TV time contributing most of the total screen time (mean = 0.83, SD = 0.70), followed by tablet time (mean = 0.16, SD = 0.33), and small amounts of time for computer (mean = 0.06, SD = 0.23), videogames (mean = 0.03, SD = 0.17), and smartphone (mean = 0.04, SD = 0.17). Overall, respondents excluded from the analyses had lower maternal educational attainment (p < 0.0001) and had more often immigrant parents (p < 0.0001). They had longer total screen time at 2 years (p < 0.0001) and 3.5 years (p < 0.0001).

**Table 1:** Comparison of sociodemographic characteristics, activities, screen viewing and drawing ability score in included and excluded respondents in the ELFE birth cohort.

	Included sample N = 7577					Excluded sample N = 4659					p^1^
	N			%		N			%		
Child sex											0.003
Boys	3798			50.1		2463			52.9		
Girls	3779			49.9		2196			47.1		
Maternal age											0.007
≤ 30 years	2171			28.7		1394			29.9		
31–40 years	4714			62.2		2707			58.1		
> 40 years	461			6.1		240			5.2		
Missing	231			3.0		318			6.8		
Gestational age at birth											< 0.0001
33–36 weeks	330			4.4		285			6.1		
≥ 37 weeks	7136			94.2		4294			92.2		
Missing	111			1.5		80			1.7		
Birth rank											0.009
First born	3380			44.6		2190			47.0		
Later born	4197			55.4		2469			53.0		
Maternal educational level											< 0.0001
Junior high school	316			4.2		275			5.9		
Technical high school	741			9.8		596			12.8		
High school graduate	1191			15.7		900			19.3		
2 years university	1838			24.3		1000			21.5		
3–4 years university	1616			21.3		777			16.7		
≥ 5 years university	1754			23.1		991			21.3		
Missing	121			1.6		120			2.6		
Paternal educational level											< 0.0001
Junior high school	441			5.8		334			7.2		
Technical high school	1135			15.0		774			16.6		
High school graduate	1399			18.5		799			17.1		
2 years university	1363			18.0		753			16.2		
3–4 years university	891			11.8		462			9.9		
≥ 5 years university	1774			23.4		930			20.0		
Missing	574			7.6		607			13.0		
Household income per consumption unit (€)											< 0.0001
< 1100	1137			15.0		824			17.7		
[1100–1670[	2761			36.4		1528			32.8		
[1670–2700[	2359			31.1		1161			24.9		
≥ 2700	836			11.0		439			9.4		
Missing	484			6.4		707			15.2		
Migration status											< 0.0001
No immigrant parent	6456			85.2		3687			79.1		
1 immigrant parent	815			10.8		653			14.0		
2 immigrant parents	296			3.9		307			6.6		
Missing	10			0.1		12			0.3		
Parents living together											0.0002
Yes	7112			93.9		4142			88.9		
No	235			3.1		198			4.2		
Missing	230			3.0		319			6.8		
Drawing/painting practice at 2 years											0.001
Never	184			2.4		140			3.0		
Sometimes	2247			29.7		1415			30.4		
Often	3087			40.7		1748			37.5		
Everyday	1783			23.5		958			20.6		
Missing	276			3.6		398			8.5		
Extra-curricular activity at 3.5 years											0.0005
Yes	1462			19.3		764			16.4		
No	6114			80.7		3793			81.4		
Missing	1			0.0		102			2.2		
Drawing execution											
Right away	6521			86.1							
Had to insist a little	599			7.9							
Had to insist a lot	390			5.1							
Missing	67			0.9							
Hand used for drawing											
Right	6440			85.0							
Left	901			11.9							
Both hands	169			2.2							
Missing	67			0.9							

Children’s activities	Missing	Mean	SD	Median	IQR	Missing	Mean	SD	Median	IQR	p
Total sleep time at 3.5 years (h/day)	574	12.30	0.81	12.32	1.03	654	12.25	0.84	12.29	1.08	0.003
Time spent playing outside at 3.5 years (h/day)	35	1.71	1.06	1.50	1.21	133	1.79	1.11	1.57	1.29	0.0002
Time between start of preschool and drawing test (months)	21	8.09	3.90	8.0	6.0	2871	7.67	4.18	7.0	6.0	0.0001
Drawing and screen time											
Child total screen time at 2 years (h/day)	539	0.77	0.90	0.52	0.84	711	0.85	1.01	0.57	0.95	< 0.0001
Child TV time at 2 years (h/day)	539	0.65	0.79	0.46	0.82	710	0.71	0.87	0.50	0.86	0.0003
Child total screen time at 3.5 years (h/day)	0	1.12	0.97	0.93	1.00	137	1.25	1.08	1.00	1.14	< 0.0001
Child TV time at 3.5 years (h/day)	20	0.83	0.70	0.64	0.81	163	0.93	0.78	0.72	0.90	< 0.0001
Child tablet time at 3.5 years (h/day)	3	0.16	0.33	0	0.17	141	0.18	0.39	0	0.24	0.0005
Child PC time at 3.5 years (h/day)	3	0.06	0.23	0	0	143	0.06	0.24	0	0	0.5
Child videogame time at 3.5 years (h/day)	3	0.03	0.16	0	0	147	0.04	0.19	0	0	0.03
Child smartphone time at 3.5 years (h/day)	8	0.04	0.17	0	0	151	0.05	0.16	0	0	0.06
Child tablet + smartphone + PC time at 3.5 years	0	0.26	0.47	0	0.33	137	0.29	0.52	0	0.40	0.0005
McCarthy drawing score	0	4.81	2.96	5.0	4.0						

*p* Chi-square test or t-test as appropriate, *IQR* interquartile range, *SD* standard deviation.

### Bivariate analyses

In bivariate analyses using zero-inflated Poisson regression, screen time in boys was associated with obtaining a null score, but not with the obtained scores above zero (Table 2). For each additional hour per day of total screen time at 2 years and at 3.5 years, boys were more likely to obtain a null drawing score (at 2 years: OR 1.08, 95% CI 1.01–1.17, at 3.5 years: OR 1.15, 95% CI 1.07–1.23). However, there was no association between the obtained drawing score above zero and screen time at 2 years (coef = 0.00, 95% CI − 0.02; 0.02) or at 3.5 years (coef = 0.001, 95% CI − 0.01; 0.02). Similar results were observed for boys’ TV time at 2 years and 3.5 years. Time spent using internet-related devices was associated with a higher likelihood to obtain a null score, in boys only (OR 1.23, 95% CI 1.07–1.41). In bivariate analyses, screen time in girls was associated both with obtaining a null score and with their above-zero score. For each additional hour per day of total screen time at 3.5 years, girls were more likely to obtain a null score (OR 1.13, 95% CI 1.03–1.24). Moreover, each additional hour of total screen time at 2 and 3.5 years was associated with a lower score above zero (at 2 years: coef − 0.02, 95% CI − 0.03; 0.00, and at 3.5 years: coef =  − 0.02, 95% CI − 0.04; − 0.01). Similar results were observed for girls’ TV and tablet time at 3.5 years.

**Table 2:** Associations of children’s screen time at 2 and 3.5 years with McCarthy drawing score at age 3.5 years, according to child sex, Elfe birth cohort.

	Boys				Girls			
	Zero-inflation modeling		Poisson-regression modeling		Zero-inflation modeling		Poisson-regression modeling	
	OR (95% CI)	p	B (95% CI)	p	OR (95% CI)	p	B (95% CI)	p
Unadjusted								
Total screen time at 2 years (h/day)	1.08 (1.01, 1.17)	0.03	0.00 (− 0.02, 0.02)	0.92	1.07 (0.95, 1.19)	0.26	− 0.02 (− 0.03, 0.00)	0.04
TV time at 2 years (h/day)	1.12 (1.03, 1.21)	0.009	0.00 (− 0.02, 0.02)	0.87	1.09 (0.96, 1.23)	0.19	− 0.01 (− 0.03, 0.00)	0.14
Total screen time at 3.5 years (h/day)	1.15 (1.07, 1.23)	< 0.0001	0.00 (− 0.01, 0.02)	0.92	1.13 (1.03, 1.24)	0.01	− 0.02 (− 0.04, − 0.01)	0.008
TV time at 3.5 years (h/day)	1.20 (1.09, 1.32)	0.0003	− 0.01 (− 0.03, 0.02)	0.63	1.22 (1.07, 1.39)	0.003	− 0.02 (− 0.04, 0.00)	0.05
Tablet time at 3.5 years (h/day)	1.04 (0.84, 1.29)	0.69	0.02 (− 0.03, 0.07)	0.39	1.08 (0.81, 1.43)	0.60	− 0.04 (− 0.09, 0.00)	0.05
Tablet + PC + smartphone time at 3.5 years (h/day)	1.23 (1.07, 1.41)	0.004	0.01 (− 0.02, 0.05)	0.48	1.07 (0.88,1.31)	0.49	− 0.03 (− 0.06, 0.00)	0.06
Model 1^1^								
Total screen time at 2 years (h/day)	1.02 (0.93, 1.11)	0.69	0.00 (− 0.02, 0.02)	0.69	1.01 (0.88, 1.16)	0.87	− 0.01 (− 0.03, 0.01)	0.50
TV time at 2 years (h/day)	1.03 (0.94, 1.14)	0.52	0.00 (− 0.02, 0.02)	0.88	1.01 (0.87, 1.18)	0.87	0.00 (− 0.02, 0.02)	0.93
Total screen time at 3.5 years (h/day)	1.04 (0.95, 1.13)	0.42	0.01 (− 0.01, 0.03)	0.53	1.07 (0.95, 1.21)	0.28	− 0.01 (− 0.03, 0.01)	0.25
TV time at 3.5 years (h/day)	1.06 (0.94, 1.20)	0.35	0.00 (− 0.02, 0.03)	0.79	1.15 (0.97, 1.35)	0.11	− 0.01 (− 0.03, 0.02)	0.51
Tablet time at 3.5 years (h/day)	0.93 (0.72, 1.20)	0.56	0.03 (− 0.02, 0.09)	0.23	1.08 (0.78, 1.50)	0.62	− 0.02 (− 0.07, 0.03)	0.47
Tablet + PC + smartphone time at 3.5 years (h/day)	1.06 (0.89, 1.27)	0.48	0.02 (− 0.02, 0.05)	0.44	1.03 (0.82, 1.30)	0.80	− 0.01 (− 0.05, 0.02)	0.46
Model 2^2^								
Total screen time at 2 years (h/day)	1.09 (1.01, 1.19)	0.03	0.00 (− 0.02, 0.02)	0.91	1.03 (0.90, 1.17)	0.69	− 0.01 (− 0.03, 0.01)	0.18
TV time at 2 years (h/day)	1.12 (1.02, 1.22)	0.02	0.00 (− 0.03, 0.02)	0.76	1.02 (0.88, 1.19)	0.74	− 0.01 (− 0.03, 0.02)	0.57
Total screen time at 3.5 years (h/day)	1.12 (1.03, 1.21)	0.005	0.00 (− 0.02, 0.02)	0.77	1.09 (0.97, 1.22)	0.14	− 0.01 (− 0.03, 0.00)	0.11
TV time at 3.5 years (h/day)	1.16 (1.04, 1.30)	0.008	0.00 (− 0.03, 0.02)	0.76	1.16 (0.99, 1.36)	0.06	− 0.01 (− 0.03, 0.01)	0.38
Tablet time at 3.5 years (h/day)	1.00 (0.79, 1.27)	0.99	0.03 (− 0.02, 0.08)	0.28	1.11 (0.81, 1.51)	0.52	− 0.03 (− 0.08, 0.02)	0.29
Tablet + PC + smartphone time at 3.5 years (h/day)	1.17 (1.00, 1.37)	0.053	0.01 (− 0.02, 0.05)	0.44	1.05 (0.84, 1.32)	0.64	− 0.02 (− 0.06, 0.01)	0.23
Model 3^3^								
Total screen time at 2 years (h/day)	1.01 (0.92, 1.11)	0.81	0.00 (− 0.02, 0.02)	0.82	1.00 (0.86, 1.15)	0.97	− 0.01 (− 0.03, 0.01)	0.53
TV time at 2 years (h/day)	1.02 (0.92, 1.13)	0.70	0.00 (− 0.02, 0.02)	0.99	0.98 (0.83, 1.16)	0.84	0.00 (− 0.02, 0.03)	0.92
Total screen time at 3.5 years (h/day)	1.01 (0.92, 1.11)	0.77	0.01 (− 0.01, 0.03)	0.49	1.05 (0.92, 1.19)	0.47	− 0.01 (− 0.03, 0.01)	0.39
TV time at 3.5 years (h/day)	1.03 (0.90, 1.17)	0.66	0.00 (− 0.03, 0.03)	0.83	1.12 (0.94, 1.34)	0.21	− 0.01 (− 0.03, 0.02)	0.67
Tablet time at 3.5 years (h/day)	0.91 (0.70, 1.19)	0.48	0.03 (− 0.02, 0.09)	0.23	1.04 (0.74, 1.46)	0.82	− 0.01 (− 0.07, 0.04)	0.62
Tablet + PC + smartphone time at 3.5 years (h/day)	1.04 (0.87, 1.25)	0.66	0.02 (− 0.02, 0.06)	0.35	1.00 (0.79, 1.28)	0.98	− 0.01 (− 0.05, 0.03)	0.56

*OR* odd-ratios, *95% CI* 95% confidence interval.

Reading: In unadjusted analyses, for each additional hour of total screen time per day at 2 years, boys were more likely to obtain a null score. However, there was no increased likelihood to obtain a higher score above zero.

^1^Model adjusted for parental educational attainment, household income, household migration status, parents living together, maternal age, birth rank, gestational age, drawing execution and hand used for drawing.

^2^Model adjusted for drawing or painting practice at 2 years (h/day), extracurricular activity at 3 years, time spent playing outside (h/day), total sleep time (h/day), time between start of kindergarten and drawing data collection (months), frequency of sedentary activities with parents, maternal age, birth rank, gestational age, drawing execution and hand used for drawing.

^3^Model adjusted for all variables in models 1 and 2.

### Multivariable analyses

In model 1, controlling for socioeconomic characteristics, we observed no significant effect of child total screen time at 2 or 3.5 years on drawing scores for boys or for girls. No significant association of device-specific screen time remained, for either boys or girls. In model 2, when controlling for child’s competing activities, boys remained more likely to obtain a null score for each additional hour of total screen time at 2 years (aOR 1.09, 95% CI 1.01–1.19) and at 3.5 years (aOR 1.12, 95% CI 1.03–1.21), with similar results for TV time and internet-related devices. However, the effect on girls’ score became unsignificant at 2 years (coef =  − 0.01, 95% CI − 0.03; 0.01) and at 3.5 years (coef =  − 0.01, 95% CI − 0.03; 0.00). When controlling for all covariates simultaneously, results were very similar to the first model controlling socioeconomic characteristics, with no significant associations between screen viewing and drawing score, either for boys or for girls.

In analyses stratified on maternal educational level, total screen time at 3.5 years was associated with a higher likelihood to have a null score, only among girls whose mother had a higher educational attainment (aOR 1.24, 95% CI 1.01–1.52) (Supplementary Table S1). Stratified analyses with other screen variables as exposure and among boys were non-significant.

## Discussion

We found that increased screen time at 2 and 3.5 years was associated with poorer drawing scores in boys and girls. However, for both sexes, these observed effects were confounded by household socioeconomic characteristics. Adjusting for the child’s competing activities altered the unadjusted associations only slightly.

In unadjusted analyses, our results are consistent with the two European studies^15,16^ that found a negative association between screen time and drawing score in children. However, we were able in our study to adjust for household socioeconomic and demographic characteristics and provide estimates not or less confounded by household factors. More broadly, in underlining the role of parental socioeconomic status in the association between screen viewing and drawing score, our findings are consistent with previous results on screen viewing and cognitive development: the context created by parents around screen viewing may better determine cognitive development outcomes^4,6^. This refers to parental behaviours largely dependent on parental socioeconomic status, specifically cultural capital: parents’ knowledge about the risks associated with screen viewing in childhood, their personal engagement in guiding their child’s activities and their own cultural practices or leisure activities may determine the ways in which they engage their child in screen viewing^17^. Moreover, when stratifying analyses on maternal educational level, we found that the negative association between total screen time at 3.5 years and drawing score remained only among girls whose mother had higher educational attainment. This suggests that in this group, screen time at 3.5 years might replace drawing time or other sedentary activities benefitting drawing skills.

Our second hypothesis was not confirmed among boys: the association between screen time and drawing score was not explained by boys’ competing activities, although effects were slightly attenuated. Among girls, although the effect size changed only slightly, association did not remain significant after adjustment for competing activities. This might suggest that girls’ competing activities mirror their household socioeconomic status, while boys’ competing activities do not. These gendered differences could also suggest that in boys, screen time displaces activities that do not promote drawing ability, while in girls, it might displace other sedentary activities that do promote drawing skills. If unstructured activity are more susceptible to displacement^28^, then we can suggest that drawing/painting practice and other sedentary activities practiced with parents are likely to be displaced by screens.

Stratifying analyses on maternal educational level, we found that the observed association between total screen time at 3.5 years and drawing score remained only among girls whose mother had higher educational attainment. This suggests that in this group, screen time might be replacing drawing time or other sedentary activities benefitting drawing skills.

It appeared that the association between screen time and drawing score played out differently between boys and girls: in boys, the associations were mainly observed with the likelihood to obtain a null score, while in girls the associations were also seen with scores above zero. This is likely to be explained by the larger share of zeros obtained by boys, and the greater variance in scores above zero among girls.

As a previous study found a negative impact of internet use on children cognitive development^29^, we tested the association of time spent using internet-related device with the outcome. The results did not differ from other screen devices. It might be that children in our sample were too young to use internet on these devices.

As stated above, the Draw-a-person testis not a measure of cognitive development per se, and can only contribute to it by measuring fine motor skills^14^. More appropriate measure of cognitive development are obtained through more comprehensive tests, such as the WPPSI, assessing all aspects of cognitive functioning^30^.

This study must be interpreted in light of its limitations. First, only children with valid data for both the exposure and outcome of interest were included in the analyses, creating a selection in our sample. However, excluded children had on average longer screen time: had they been included, and given our findings, the associations we found would have been strengthened. Second, the large number of zeros obtained in our sample suggests that children aged 3.5 years might be too young for the Draw-a-Person test. At this age in our population, the test has only a limited ability to discriminate between more or less elaborate drawings, as many children fail to produce a discernible person, or to follow the rule to draw a person. Because drawing ability wasn’t measured at 2 years, we could not entirely reject the hypothesis of reverse causation, i.e. that cognitive functioning at 2 years (measured through poor drawing ability) determined longer screen time at 3.5 years. However, and given the poor results of children in the Drawing test displayed in this analysis at 3.5 years, we may question the relevance and feasibility of measuring drawing capacity before 3 years old. We can’t exclude the hypothesis that poor levels of capacities needed for the development of drawing skills, such as attention, visual-motor coordination or working memory, at an early age, might lead to longer screen time. Moreover, child screen time was collected from parents and may be subject to under-reporting. However, if all parents under-reported screen time to the same extent, it would not alter our associations. Although it has been argued that parents’ estimation of their child’s screen time is only moderately correlated with actual screen time^31,32^, the Elfe questionnaire used detailed recall periods (weekdays, Saturdays, Sundays) and different types of screen devices, guiding parental recall and limiting bias. Additionally, parents may have had difficulties accurately assessing children’s competing activities, possibly suffering from more bias than socioeconomic and demographic variables. Unlike all other covariates collected at 3.5 years, frequency of drawing/painting was measured at 2 years. While we assumed that it provided a reliable estimation of drawing/painting practice at 3.5 years, drawing practice at 2 years may have been replaced by screen time or other activities at 3.5 years. However, even over-reported, drawing practice only partly confounded our association of interest. Moreover, without information collected on the context of screen viewing (e.g. parental presence, discussion of content with an adult) or media content, we could not take these elements into consideration to refine our analysis of screen viewing. For instance, age-appropriate educational programs, discussed with an adult, possibly enhancing language development, may impact positively cognitive development and drawing ability. Lastly, we could not account for the potentially confounding effect of children or parental well-being: we can hypothesize that maternal or paternal depression, for instance, might determine longer screen time for the child and have a deleterious effect on child cognitive development.

Our main strength was the use of data from a nationally representative birth cohort with a very large sample size. Although 38% of the total sample at 3.5 years were excluded from our analyses for lack of data on drawing score or screen time, excluded respondents had on average longer screen time, and more unfavourable social characteristics, prompting us to hypothesize lower drawing scores among them. Therefore, their exclusion may have led to an underestimation of the strengths of the associations. Additionally, we were able to measure screen viewing at two time points—2 years and 3.5 years, with findings consistent across models. Finally, using a wide range of household characteristics, we were able to accurately assess the association between screen time and drawing ability, taking into account household socioeconomic status.

Our study shows that among young children, differences in drawing scores according to screen time were small before adjusting for SES and negligible after adjustment, seriously questioning previous findings. Our findings underline the importance of considering parents’ social position when measuring the potential effect of screen viewing on child development. Screen viewing in itself is unlikely to be responsible for poor drawing ability at 3.5 years. Research at later ages are warranted to investigate further hypotheses, such as whether early screen viewing has delayed or cumulative effects on drawing ability over time.

### Supplementary Information

Supplementary Information.

## Data Availability

The data necessary to reproduce the analyses presented here are not publicly accessible for reasons of privacy for the participants. Established researchers who would like access to the data from the Elfe cohort study can request them to the Committee of Access to the Data from the Elfe cohort on the website of the survey: www.elfe-france.fr. The analytic code necessary to reproduce the analyses presented in this paper is available from the corresponding author upon reasonable request. The analyses presented here were not preregistered.
