# Supplementary material for: Associations between screen viewing at 2 and 3.5 years and drawing ability at 3.5 years among children from the French nationwide Elfe birth cohort
Source: Sci Rep. 2024 Jan 3;14:348. doi: 10.1038/s41598-023-50767-0 (PMC10764867; doi:10.1038/s41598-023-50767-0)

Supplementary information

**Title**: Associations between screen viewing at 2 and 3.5 years and drawing ability at 3.5 years among children from the French nationwide Elfe birth cohort

**Authors**: Lorraine Poncet, Mélèa Saïd, Shuai Yang, Falk Müller-Riemenschneider, Claire Berticat, Michel Raymond, Mélissa Barkat-Defradas, Marie-Aline Charles, Jonathan Y. Bernard^*^

Supplementary table S1 – Association of screen time at 2 and 3.5 years and McCarthy drawing score, stratified on maternal educational level, zero-inflated Poisson regression

|  |  | **Boys** | | | |  | **Girls** | | | |
| --- | --- | --- | --- | --- | --- | --- | --- | --- | --- | --- |
|  |  | **Zero-inflation modeling** | | **Poisson-regression modeling** | |  | **Zero-inflation modeling** | | **Poisson-regression modeling** | |
|  | **N** | **OR (95% CI)** | **p** | **B (95% CI)** | **p** | **N** | **OR (95% CI)** | **p** | **B (95% CI)** | **p** |
| Total screen time at 2 y (h/day) |  |  |  |  |  |  |  |  |  |  |
| Below high school | 450 | 1.13 (0.98, 1.31) | 0.09 | 0.00 (-0.04, 0.05) | 0.83 | 475 | 0.88 (0.70, 1.12) | 0.30 | 0.01 (-0.03, 0.04) | 0.61 |
| ≤ 2 years university | 1403 | 1.03 (0.92, 1.15) | 0.63 | 0.00 (-0.02, 0.03) | 0.80 | 1386 | 1.10 (0.92, 1.33) | 0.42 | -0.01 (-0.04, 0.02) | 0.42 |
| ≥ 3 years university | 1587 | 1.07 (0.90, 1.26) | 0.45 | 0.00 (-0.04, 0.03) | 0.78 | 1558 | 1.05 (0.83, 1.33) | 0.68 | -0.03 (-0.06, 0.01) | 0.14 |
| TV time at 2 y (h/day) |  |  |  |  |  |  |  |  |  |  |
| Below high school | 450 | 1.13 (0.96, 1.33) | 0.13 | 0.00 (-0.04, 0.05) | 0.93 | 475 | 0.92 (0.71, 1.18) | 0.50 | 0.01 (-0.03, 0.05) | 0.54 |
| ≤ 2 years university | 1403 | 1.06 (0.93, 1.21) | 0.36 | 0.00 (-0.03, 0.03) | 0.81 | 1386 | 1.10 (0.89, 1.36) | 0.39 | -0.01 (-0.04, 0.02) | 0.60 |
| ≥ 3 years university | 1587 | 1.09 (0.89, 1.33) | 0.42 | -0.01 (-0.05, 0.03) | 0.57 | 1558 | 1.08 (0.82, 1.43) | 0.57 | -0.01 (-0.05, 0.03) | 0.52 |
| Total screen time at 3.5 y (h/day) |  |  |  |  |  |  |  |  |  |  |
| Below high school | 475 | 1.11 (0.96, 1.28) | 0.16 | 0.00 (-0.04, 0.04) | 0.82 | 495 | 0.97 (0.80, 1.18) | 0.77 | 0.01 (-0.02, 0.04) | 0.59 |
| ≤ 2 years university | 1445 | 1.07 (0.95, 1.21) | 0.25 | 0.01 (-0.01, 0.04) | 0.37 | 1440 | 1.05 (0.88, 1.26) | 0.56 | -0.02 (-0.04, 0.01) | 0.21 |
| ≥ 3 years university | 1626 | 1.10 (0.94, 1.28) | 0.22 | -0.01 (-0.04, 0.02) | 0.57 | 1602 | 1.24 (1.01, 1.52) | 0.03 | -0.03 (-0.06, 0.00) | 0.08 |
| TV time at 3.5 y (h/day) |  |  |  |  |  |  |  |  |  |  |
| Below high school | 470 | 1.17 (0.95, 1.43) | 0.13 | -0.01 (-0.07, 0.04) | 0.65 | 494 | 1.24 (0.95, 1.62) | 0.11 | 0.03 (-0.02, 0.08) | 0.23 |
| ≤ 2 years university | 1444 | 1.09 (0.92, 1.29) | 0.29 | 0.01 (-0.03, 0.05) | 0.70 | 1438 | 1.05 (0.83, 1.35) | 0.66 | -0.01 (-0.05, 0.02) | 0.40 |
| ≥ 3 years university | 1623 | 1.08 (0.88, 1.33) | 0.44 | -0.02 (-0.06, 0.02) | 0.37 | 1601 | 1.16 (0.87, 1.55) | 0.30 | -0.03 (-0.07, 0.01) | 0.14 |
| Tablet time at 3.5 y (h/day) |  |  |  |  |  |  |  |  |  |  |
| Below high school | 474 | 0.99 (0.63, 1.55) | 0.94 | 0.03 (-0.08, 0.14) | 0.59 | 495 | 0.76 (0.43, 1.35) | 0.35 | 0.00 (-0.08, 0.09) | 0.96 |
| ≤ 2 years university | 1445 | 0.96 (0.68, 1.34) | 0.79 | 0.05 (-0.02, 0.12) | 0.16 | 1440 | 1.14 (0.71, 1.82) | 0.59 | -0.05 (-0.13, 0.02) | 0.18 |
| ≥ 3 years university | 1626 | 1.09 (0.71, 1.68) | 0.68 | 0.01 (-0.08, 0.10) | 0.79 | 1601 | 1.44 (0.83, 2.48) | 0.19 | -0.03 (-0.12, 0.06) | 0.50 |
| Tablet+PC+smartphone time at 3.5 y (h/day) |  |  |  |  |  |  |  |  |  |  |
| Below high school | 475 | 1.25 (0.93, 1.67) | 0.13 | 0.02 (-0.06, 0.10) | 0.67 | 495 | 0.67 (0.43, 1.04) | 0.07 | 0.00 (-0.05, 0.06) | 0.89 |
| ≤ 2 years university | 1445 | 1.06 (0.84, 1.34) | 0.63 | 0.04 (-0.01, 0.09) | 0.16 | 1440 | 1.14 (0.81, 1.60) | 0.46 | -0.03 (-0.08, 0.03) | 0.31 |
| ≥ 3 years university | 1626 | 1.25 (0.93, 1.67) | 0.13 | -0.01 (-0.07, 0.05) | 0.77 | 1602 | 1.44 (0.99, 2.09) | 0.06 | -0.03 (-0.10, 0.03) | 0.32 |

Reading: For each additional hour of total screen time per day at 3.5 years, girls with highest maternal educational level were more likely to obtain a null score. However, there was no increased likelihood to obtain a higher score above zero.

Analyses stratified on maternal educational level, adjusted for maternal age, birth rank, gestational age, drawing execution and hand used for drawing.

Supplementary Figure S1 – Distribution of the McCarthy drawing score in the Elfe birth cohort at 3.5 years


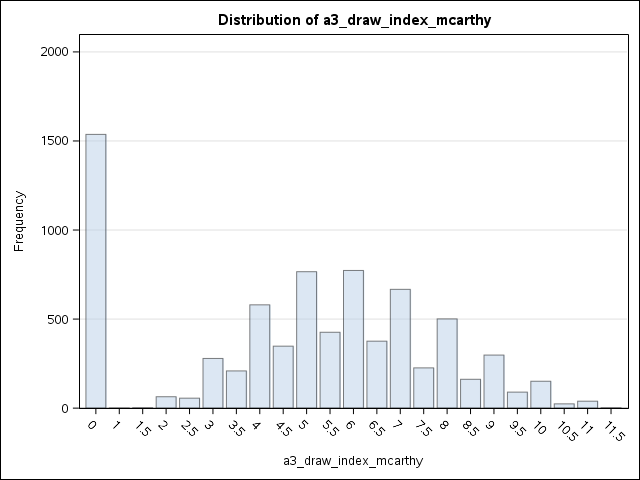

Supplement: Supplementary file 1 — Supplementary Information. [file 41598_2023_50767_MOESM1_ESM.docx]
